# Global Trends of Stem Cell Precision Medicine Research (2018–2022): A Bibliometric Analysis

**DOI:** 10.3389/fsurg.2022.888956

**Published:** 2022-06-23

**Authors:** Muge Liu, Fan Yang, Yingbin Xu

**Affiliations:** Department of Burn Surgery, The First Affiliated Hospital of Sun Yat-Sen University, Guangzhou, China

**Keywords:** induced pluripotent stem cells (iPSCs), precision medicine, personalized medicine, organoids, bibliometric analysis

## Abstract

**Background:**

Stem cells are a group of cells that can self-renew and have multiple differentiation capabilities. Shinya Yamanaka first discovered a method to convert somatic cells into pluripotent stem cells in 2006. Stem cell therapy can be summarized into three aspects (regenerative treatment, therapy targeted at stem cells, and establishment of disease models). Disease models are mainly established by induced pluripotent stem cells, and the research of stem cell precision medicine has been promising in recent years. Based on the construction of 3D, patient-specific disease models from pluripotent induced stem cells, proper research on disease development and treatment prognosis can be realized. Bibliometric analysis is an efficient way to quickly understand global trends and hotspots in this field.

**Methods:**

A literature search of stem cell precision medicine research from 2018 to 2022 was carried out using the Web of Science Core Collection.VOSviewer, R-bibliometrix, and CiteSpace software programs were employed to perform the bibliometric analysis.

**Results:**

A total of 552 publications were retrieved from 2018 to 2022. Annual publication outputs trended upward and reached a peak of 172 in 2021. The United States contributed the most publications (160, 29.0%) to the field, followed by China (63, 11.4%) and Italy (60, 10.9%). International academic collaborations were active. CANCERS was considered the most productive journal with 18 documents. NATURE was the most co-cited journal with 1860 times citations. The most cited document was entitled “Induced Pluripotent Stem Cells for Cardiovascular Disease Modeling and Precision Medicine: A Scientific Statement From the American Heart Association” with 9 times local citations. “ precision medicine” (*n* = 89, 12.64%), “personalized medicine” (*n* = 72, 10.23%), “stem cells” (*n *= 43, 4.40%), and “induced pluripotent stem cells” (*n* = 41, 5.82%), “cancer stem cells” (*n* = 31, 4%), “organoids” (*n* = 26, 3.69%) were the top 6 frequent keywords.

**Conclusion:**

The present study performs a comprehensive investigation concerning stem cell precision medicine (2018–2022) for the first time. This research field is developing, and a deeper exploration of 3D patient-specific organoid disease models is worth more research in the future.

## Introduction

Stem cells are undifferentiated or partially differentiated cells with the ability to self-renew and multiple differentiation (1). Fertilized eggs belong to totipotent stem cells. Pluripotent stem cells exist in blastocysts with 50–150 cells in mammals and differentiate into all cell types required by the body during growth and development, called embryonic stem cells (2). Adult stem cells, including hematopoietic stem cells, basal skin cells, and mesenchymal stem cells, are located in specific ecological niches in the body, like bone marrow and gonads, which can differentiate into several kinds of somatic cells (3). Stem cells can be classified as totipotent stem cells (fertilized eggs), pluripotent stem cells (embryonic stem cells), multipotent stem cells (mesenchymal stem cells), and unipotent stem cells (basal cells) by the differentiation potency.

Somatic cell nuclear transfer is the early way to create pluripotent stem cells, by which the cloned sheep, Dolly, was born (4). Shinya Yamanaka pioneered the development of induced pluripotent stem cells in 2006, using a combination of Oct3/4, Sox2, c-Myc, and Klf4 to convert mouse fibroblasts into pluripotent stem cells (5). The same chemical factors successfully transformed human fibroblasts into pluripotent stem cells (6). Junying Yu et al. optimized the combination from Oct3/4, Sox2, c-Myc, Klf4 to Oct4, Sox2, NANOG, LIN28, and successfully produced pluripotent stem cells in 2007 (7). The clinical application of stem cells can be summarized in three aspects: regenerative treatment, therapy targeted at stem cells, and disease model for research and drug development.

Using stem cells to replace the body’s dysfunctional tissue has been studied since 1963 when bone-marrow cells were reported to have saved an animal from lethal radiation. Embryonic stem cells helped treat retinal diseases (8), Parkinson’s disease (9), Huntington’s disease (10), spinal cord injury (11), myocardial infarction (12), and type 1 diabetes (13–15) in preclinical studies. In clinical studies, embryonic stem cells have been used for cell therapy in patients with retinal diseases, and severe ischemic left ventricular dysfunction (16–20), which is safe in the medium and long term. The iPSC was transplanted into animal models for treating Parkinson’s disease (21), Huntington’s disease (22), Duchenne muscular dystrophy (23), Fanconi anemia (24), and hemophilia A (25) in preclinical studies. In clinical studies, induced pluripotent stem cells were also used to treat retinal diseases, but the efficacy and safety were uncertain (26). Besides self-renewal and differentiation, stem cells can regulate the activities of surrounding cells by paracrine factors. However, due to the high variability of the stem cell population, the regulatory mechanism has not been clarified. Another strategy of stem cell therapy is to activate the proliferation and differentiation potential of somatic stem cells, such as altering erythropoietin progenitor cell activity. Specific disease models can be constructed by stem cells, which are promising in disease research and new drug development. These disease models include tumors, cardiovascular disease, and nervous system disease. These disease models are mainly completed by induced pluripotent stem cells.

Precision medicine (27) is an emerging medical model which provides personalized medical diagnosis, treatment, and care for specific patients after considering their genetic information, physiological/pathological information, living environment, and many other aspects. Compared with the traditional one-size-fits-all approach, more attention is paid to the influence of individual differences on disease development. The research on stem cell precision medicine shows a significant development in recent years. Stem cell technology, especially induced pluripotent stem cell technology, gradually matures. Researchers can establish disease/patient-specific models to accurately reflect human disease mechanisms and the individual difference in drug responses based on the pluripotent and reproducible human stem cells. The realization of precision medicine is an inherent advantage that traditional disease models cannot obtain. Disease models have been established using induced pluripotent stem cells by the clustered regularly interspaced short palindromic repeats (CRISPR)/CRISPR-associated system (CRISPR-Cas9) genome engineering tool, including cardiomyopathy, valvular disease, primary microencephaly, cystic liver fibrosis, colorectal cancer, etc. Nevertheless, stem cell-based disease models are still in the early stages of development. 3D organoid models can accurately simulate organ morphology, form tissue-like architecture, and realize organ/disease development in vitro organ culture system. More complex 3D organoid multisystem models are still in development, including the addition of circulatory systems and lymphatic systems.

As an effective confirmed method of analyzing the dynamics and trends of research in a specific field, bibliometrics has rapidly developed in past decades (28). Unlike the conventional measurement of scientific documents, bibliometrics is a prevailing tool that can comprehensively investigate existing studies in a specific field. It can be easily employed with big data accessible to the public, such as Web of Science, PubMed, and MEDLINE. Statistical tools, including R-bibliometrix, VOSviewer, CiteSpace, BICOMB, and BibExcel, are integrated as an automatic workflow to carry out multistep bibliometric analyses. In prior studies, bibliometric analysis has been successfully performed in various disciplines, especially in environmental science and medicine (29).

In this study, we aim to explore the following objects in the field of stem cell precision medicine: 1. productivity of publications; 2. productive countries/regions; 3. leading affiliations; 4. influential journals; 5. key documents/references; and 6. key words. With the technology of bibliometric analysis, we can conduct a quick review of the research process of stem cell precision medicine in the last five years to provide reference value to clinicians and scholars in future studies.

## Method

### Data Collection

We carried out the Web of Science database to conduct a comprehensive literature retrieval for 2018 to 2022. The process was limited within the Web of Science Core Collection, and no restriction on the edition. The retrieval strategy was as follows: Topic =(“ stem cell*” or “pluripotent stem cell*” or “PSC*” or “MSC*” or “iPSC*”). Related Documents, including” articles” and “reviews”, were selected. All search was performed on a single day, January 22, 2022. Documents were downloaded as original data from WoS for further bibliometric analysis.

### Statistical Analysis

Indicators, including the annual publications, countries, affiliations, and languages, were extracted from the WoS and managed using Microsoft Excel for Mac 2018 edition 16.18 (181014). The R-bibliometrix was performed to sort the countries and regions, institutions, individuals, and citations in the order of publications productivity. Impact Factor was obtained from WoS and consistent with the latest vision of Journal Citation Reports 2020 as a measurement of journal analysis. To better understand the academic relationships among different countries, we used VOSviewer (1.6.16) to visualize the intensity of collaborations with the lines connecting countries. The “treemap” function of the R-bibliometrics was employed to sort the author’s keywords in the order of frequency. CiteSpace (5.7.R5) was used to conduct the burst of reference citations from 2018 to 2022, and the minimum duration of burst was set for one year.

## Results

### Annual Output

**Figure 1:**
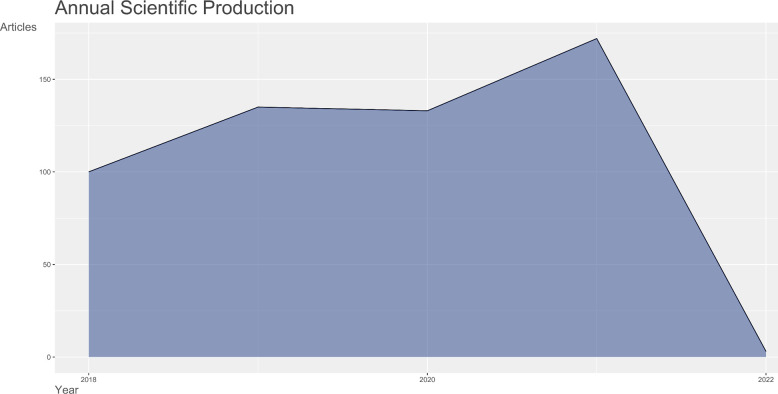
Annul number of publications in the field of stem cell precision medicine (2018–2022).

The total number of publications on stem cell precision medicine research was 552 from 2018 to 2022, of which articles and reviews accounted for 280 (50.72%) and 272 (49.28%), respectively. The languages of publications were mainly English 544 (98.55%) and other languages including French, German, etc. The annual outputs of stem cell precision medicine publications are shown in Figure 1. This period’s continuously increasing trends were shown with the peak point at 2021 of 172.

### Country/Region Analysis

**Figure 2:**
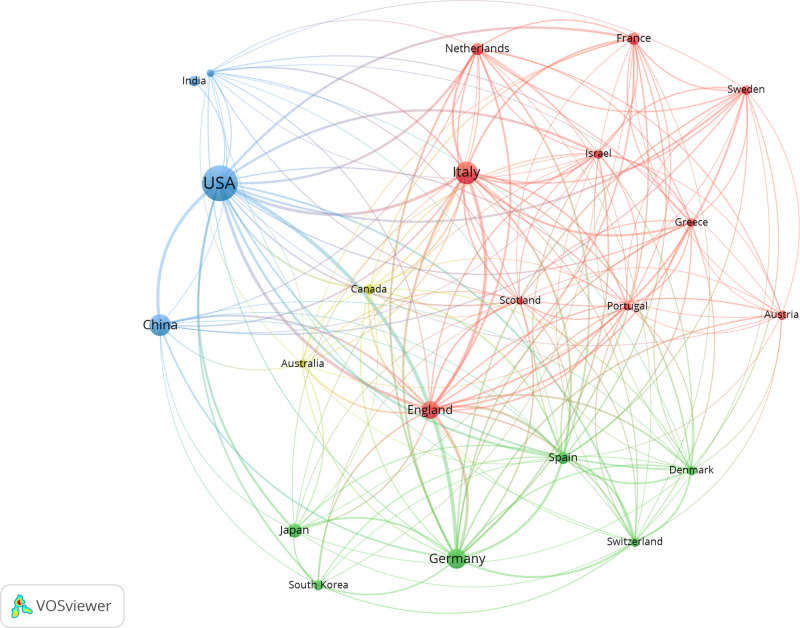
Geographical distribution and collaborations among countries in stem cell precision medicine. The circles represent countries, with the larger circle corresponding to more publications. The connecting lines represent the academic cooperation between countries, with the thicker line corresponding to closer cooperation. Different colors represent different clusters automatically calculated by VOSviewer.

We identified 50 countries that contributed to stem cell precision medicine research. The top 10 productive countries were selected and presented in Table 1. The United States was the most productive country (*n* = 160), followed by China (*n* = 63), Italy (*n* = 60), Germany (*n* = 38), and the UK (*n* = 22). In Figure 2, the larger circle represented more publications, and the thicker line represented more collaborations. Active collaborations were observed, as the United States had active academic exchanges with many countries, including Italy, China, Germany, England, Japan, India, and so on.

**Table 1:** The top 10 most productive countries of stem cell precision medicine research.

Country	Articles	SCP	MCP	MCP_Ratio
USA	160 (28.99%)	124	36	0.225
CHINA	63 (11.41%)	50	13	0.2063
ITALY	60 (10.87%)	43	17	0.2833
GERMANY	38 (6.88%)	20	18	0.4737
UNITED KINGDOM	22 (3.99%)	11	11	0.5
JAPAN	21 (3.80%)	19	2	0.0952
FRANCE	16 (2.90%)	12	4	0.25
INDIA	15 (2.72%)	13	2	0.1333
CANADA	12 (2.17%)	9	3	0.25
KOREA	12 (2.17%)	10	2	0.1667

*SCP, intra-country collaboration; MCP, inter-country collaboration.*

The top 10 productive affiliations worldwide are listed in Table 2. Among these affiliations, seven were located in America, while the rest three were located in Austria, Canada, and Italy. The top 3 productive affiliations were Stanford University, Medical University of Vienna, and Harvard Medical School, which respectively contributed 42, 30, and 29 pieces of research on stem cell precision medicine.

**Table 2:** The top 10 most productive affiliations of stem cell precision medicine research.

Affiliations	Articles	Nation
Stanford University	42	USA
Medical University of Vienna	30	Austria
Harvard Medical School	29	USA
Icahn School of Medicine at Mount Sinai	24	USA
University of Pennsylvania	22	USA
University of Toronto	19	Canada
University of Illinois Chicago	17	USA
University of Pittsburgh	17	USA
University of Washington	17	USA
University of Milan	16	Italy

### Journal Analysis

We identified 344 journals associated with publications of stem cell precision medicine from 2018 to 2022. The journals with more than 10 publications in this field are shown in Table 3. As of January 2022, the top 3 productive journals were CANCERS, CELLS, and INTERNATIONAL JOURNAL OF MOLECULAR SCIENCES, with 18, 15, and 14 publications. The journals with the three highest impact factors were BIOMATERIALS, EBIOMEDICINE, FRONTIERS IN CELL AND DEVELOPMENTAL BIOLOGY, which published 5, 5, and 6 articles.

**Table 3:** The top 10 most productive journals of stem cell precision medicine research.

Sources	Articles	IF2020	JCR2020
CANCERS	18	6.639	Q1 (ONCOLOGY)
CELLS	15	6.600	Q2 (CELL BIOLOGY)
INTERNATIONAL JOURNAL OF MOLECULAR SCIENCES	14	5.924	Q1 (BIOCHEMISTRY & MOLECULAR BIOLOGY) Q2 (CHEMISTRY, MULTIDISCIPLINARY)
JOURNAL OF PERSONALIZED MEDICINE	9	4.945	Q1 (HEALTH CARE SCIENCES & SERVICES) Q1 (MEDICINE, GENERAL & INTERNAL)
SCIENTIFIC REPORTS	7	4.380	Q1 (MULTIDISCIPLINARY SCIENCES)
FRONTIERS IN CELL AND DEVELOPMENTAL BIOLOGY	6	6.684	Q1 (DEVELOPMENTAL BIOLOGY) Q2 (CELL BIOLOGY)
JOURNAL OF CLINICAL MEDICINE	6	4.242	Q1 (MEDICINE, GENERAL & INTERNAL)
BIOMATERIALS	5	12.479	Q1 (ENGINEERING, BIOMEDICAL) Q1 (MATERIALS SCIENCE, BIOMATERIALS)
EBIOMEDICINE	5	8.143	Q1 (MEDICINE, RESEARCH & EXPERIMENTAL)
FRONTIERS IN BIOENGINEERING AND BIOTECHNOLOGY	5	5.890	Q1 (MULTIDISCIPLINARY SCIENCES)

The term co-citation refers to a situation where two documents both appear in the reference of the third document. A total of 10 journals had been co-cited no less than 570 times (T = 570), as shown in Table 4. NATURE had the highest co-citation frequency of 1,860, followed by CELL and J Cell Stem Cell, which had been co-cited up to 1,241 and 1,218 times, respectively. Scientific Reports was both in the top 10 productive and co-cited journals.

**Table 4:** The 10 most co-cited journals of stem cell precision medicine research from 2018 to 2022.

Sources	Citation	IF2020	JCR2020
NATURE	1,860	49.962	Q1 (MULTIDISCIPLINARY SCIENCES)
CELL	1,241	41.584	Q1 (BIOCHEMISTRY & MOLECULAR BIOLOGY) Q1 (CELL BIOLOGY)
Cell Stem Cell	1,218	24.633	Q1 (CELL & TISSUE ENGINEERING) Q1 (CELL BIOLOGY)
PROCEEDINGS OF THE NATIONAL ACADEMY OF SCIENCES OF THE UNITED STATES OF AMERICA	1,120	11.205	Q1 (MULTIDISCIPLINARY SCIENCES)
BLOOD	1,080	23.629	Q1 (HEMATOLOGY)
SCIENCE	986	47.728	Q1 (MULTIDISCIPLINARY SCIENCES)
PLoS One	903	3.24	Q2 (MULTIDISCIPLINARY SCIENCES)
Scientific Reports	884	4.38	Q1 (MULTIDISCIPLINARY SCIENCES)
NEW ENGLAND JOURNAL OF MEDICINE	686	91.253	Q1 (MEDICINE, GENERAL & INTERNAL)
Nature Communications	593	14.919	Q1 (MULTIDISCIPLINARY SCIENCES)

**Figure 3:**
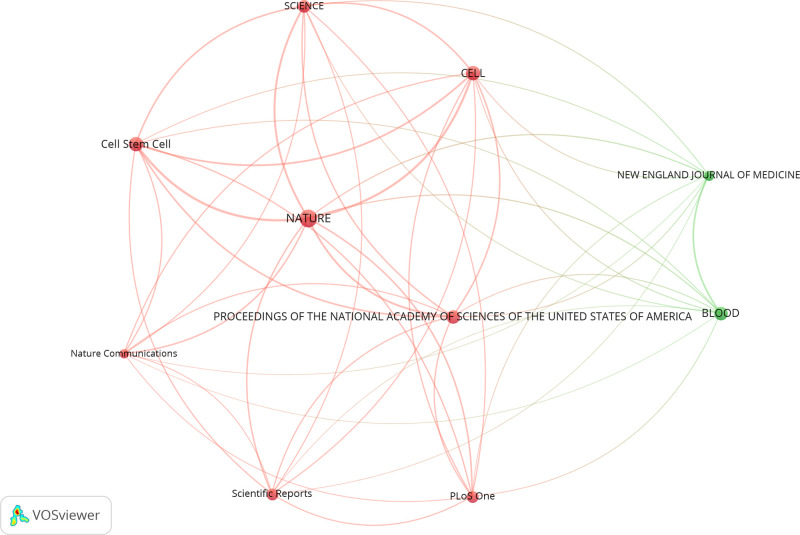
Co-citation relationships among journals related to stem cell precision medicine research. The circles represent journals, with the larger circle corresponding to more citations. The connecting lines represent the co-citation relationship between journals, with the thicker line corresponding to more co-citations. Different colors represent different clusters automatically calculated by VOSviewer.

In VOSviewer, we created a map to reflect the co-citation relationships among journals whose publications had been at least co-cited 570 times (Figure 3). NATURE was represented by the giant circle in the center and had active co-citation relationships with other journals, especially with CELL, Cell Stem Cell, PROCEEDINGS OF THE NATIONAL ACADEMY OF SCIENCES OF THE UNITED STATES OF AMERICA, and so on.

### Document Analysis

The top 10 documents in terms of local citation times are listed in Table 5. The most cited document was entitled “Induced Pluripotent Stem Cells for Cardiovascular Disease Modeling and Precision Medicine: A Scientific Statement From the American Heart Association” on CIRCULATION-GENOMIC AND PRECISION MEDICINE, which was published by Musunuru K in 2018 with 9 local citations. The second was “Disease Modeling Using 3D Organoids Derived from Human Induced Pluripotent Stem Cells” on INTERNATIONAL JOURNAL OF MOLECULAR SCIENCES, published by Ho BX in 2018 with 7 local citations. The third was “Organs-on-a-Chip: A Fast Track for Engineered Human Tissues in Drug Development” on CELL STEM CELL, published by Ronaldson-Bouchard K in 2018 with 6 local citations.

**Table 5:** The 10 most cited documents of stem cell precision medicine in the past 5 years.

Document	First author	Journal	Year	Local Citations
Induced Pluripotent Stem Cells for Cardiovascular Disease Modeling and Precision Medicine: A Scientific Statement From the American Heart Association	Musunuru K	CIRCULATION-GENOMIC AND PRECISION MEDICINE	2018	9
Disease Modeling Using 3D Organoids Derived from Human Induced Pluripotent Stem Cells	Ho BX	INTERNATIONAL JOURNAL OF MOLECULAR SCIENCES	2018	7
Organs-on-a-Chip: A Fast Track for Engineered Human Tissues in Drug Development	Ronaldson-Bouchard K	CELL STEM CELL	2018	6
Human iPSC banking: barriers and opportunities	Huang CY	JOURNAL OF BIOMEDICAL SCIENCE	2019	6
Patient and Disease-Specific Induced Pluripotent Stem Cells for Discovery of Personalized Cardiovascular Drugs and Therapeutics	Paik DT	PHARMACOLOGICAL REVIEWS	2020	6
Enhanced Utilization of Induced Pluripotent Stem Cell-Derived Human Intestinal Organoids Using Microengineered Chips	Workman MJ	CELLULAR AND MOLECULAR GASTROENTEROLOGY AND HEPATOLOGY	2018	5
Human iPSC-Derived Blood-Brain Barrier Chips Enable Disease Modeling and Personalized Medicine Applications	Vatine GD	CELL STEM CELL	2019	5
Personalised organs-on-chips: functional testing for precision medicine	van den Berg A	LAB ON A CHIP	2019	5
Personalized medicine in cardio-oncology: the role of induced pluripotent stem cell	Sayed N	CARDIOVASCULAR RESEARCH	2019	5
Towards Precision Medicine With Human iPSCs for Cardiac Channelopathies	Wu JC	CIRCULATION RESEARCH	2019	5

**Figure 4:**
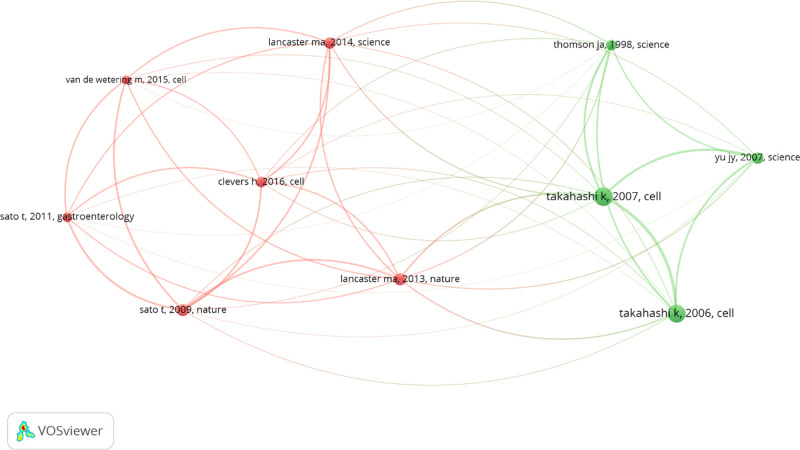
The co-citation relationships among references (T > 28) of stem cell precision medicine (2018–2022). The circles represent references, with the larger circle corresponding to more citations. The connecting lines represent the co-citation relationship between references, with the thicker line corresponding to more co-citations. Different colors represent different clusters automatically calculated by VOSviewer.

The top 10 references with the highest co-citations are listed in Table 6. “Induction of pluripotent stem cells from adult human fibroblasts by defined factors” had the most co-citations (*n* = 91), followed by “Induction of pluripotent stem cells from mouse embryonic and adult fibroblast cultures by defined factors” (*n* = 84) and “Cerebral organoids model human brain development and microcephaly” (*n* = 46). The top 10 references had at least over 28 co-citations (T = 28) and a map constructed in the VOSveiwer, to present the co-citation relationships between the references (Figure 4). The top co-cited references tended to have co-citation relationships according to the thickness of lines between them.

**Table 6:** The 10 most co-cited references of stem cell precision medicine in the past 5 years.

Document	First author	Journal	Year	Co-Citations
Induction of pluripotent stem cells from adult human fibroblasts by defined factors	Takahashi K	CELL	2007	91
Induction of pluripotent stem cells from mouse embryonic and adult fibroblast cultures by defined factors	Takahashi K	CELL	2006	84
Cerebral organoids model human brain development and microcephaly	Lancaster MA	NATURE	2013	46
Single Lgr5 stem cells build crypt-villus structures in vitro without a mesenchymal niche	Sato T	NATURE	2009	43
Induced pluripotent stem cell lines derived from human somatic cells	Yu J	SCIENCE	2007	40
Organogenesis in a dish: modeling development and disease using organoid technologies	Lancaster MA	SCIENCE	2014	38
Embryonic stem cell lines derived from human blastocysts	Thomson JA	SCIENCE	1998	36
Modeling Development and Disease with Organoids	Clevers H	CELL	2016	33
Long-term expansion of epithelial organoids from human colon, adenoma, adenocarcinoma, and Barrett’s epithelium	Sato T	GASTROENTEROLOGY	2011	30
Prospective derivation of a living organoid biobank of colorectal cancer patients	van de Wetering M	CELL	2015	28

### Key Words Analysis

Keywords were set as “author keywords” in the VOSviewer, and the top 50 keywords were automatically listed in terms of frequencies (sizes of cubes) in Figure 5A. We found that the top 50 keywords with frequency rates higher than 3% account for 12% and they were “precision medicine” (*n* = 89, 12.64%), “personalized medicine” (*n* = 72, 10.23%), “stem cells” (*n* = 43, 4.40%), “induced pluripotent stem cells” (*n* = 41, 5.82%), “ cancer stem cells” (*n* = 31, 4%), and “organoids” (*n* = 26, 3.69%). A map (Figure 5B) was built to reflect the co-occurrence relationships between high-frequency keywords. “stem cells” “induced pluripotent stem cells” “cancer stem cells” “organoids” and “regenerative medicine” had strong co-occurrence relationships with “precision medicine” and “personalized medicine”, which reflected the classic research direction and basic theory of stem cell precision medicine research.

**Figure 5:**
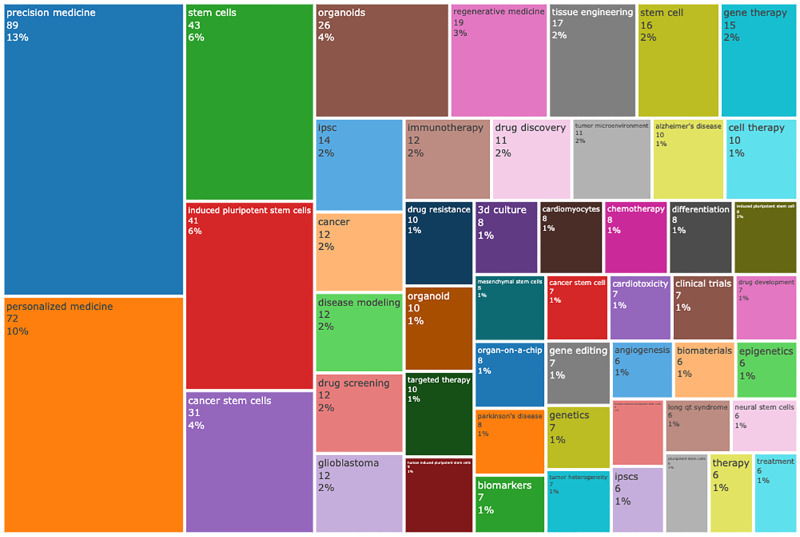
(Continued)

**Figure 5:**
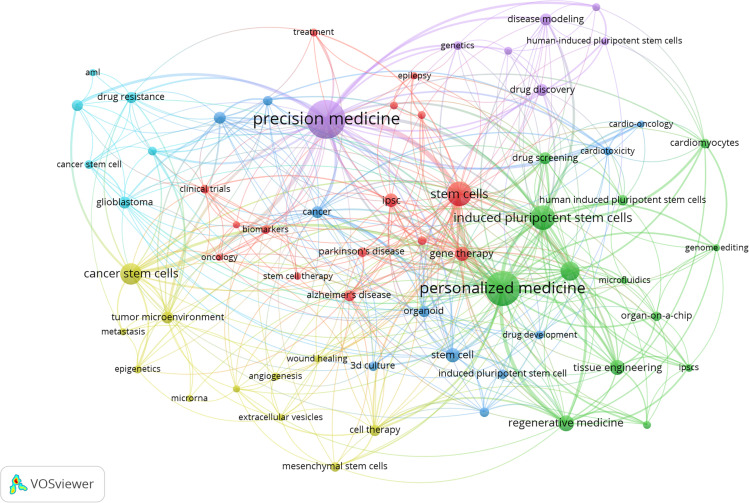
The keyword distrbution of stem cell precision medicine (A) and the co-occurrence relationships among keywords (B). In Figure 5B, the circles represent keywords, with the larger circle corresponding to more occurrences. The connecting lines represent the co-occurrence relationship between keywords, with the thicker line corresponding to more co-occurrences. Different colors represent different clusters automatically calculated by VOSviewer.

### Burst Analysis

**Figure 6:**
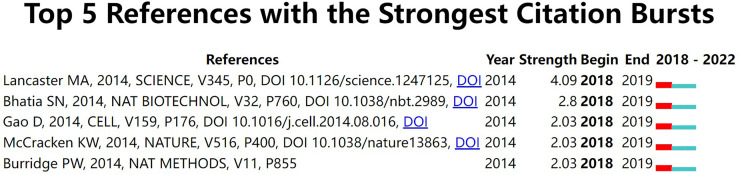
The top 5 references with citation burst during the last 5 years. “Year” represents the publication year. “Strength” represents the burst strength. “Begin” and “End” represent the beginning year and ending year.

With CiteSpace, we performed a burst analysis of reference citations, and five references were selected during the period. As shown in Figure 6, the reference entitled “Organogenesis in a dish: modeling development and disease using organoid technologies” had the highest burst strength (4.09), followed by “Microfluidic organs-on-chips” (2.8) and “Organoid cultures derived from patients with advanced prostate cancer” (2.03).

## Discussion

### Research Status and Trends

We performed a worldwide investigation of the literature on stem cell precision medicine from WoSCC from 2018 to 2022 by bibliometric analysis. Each year’s total output of publications can reflect academics’ overall development and attention in a specific research field. The general trend of research output regarding stem cell precision medicine was rising. A peak was reached in 2021, suggesting that this field had been a subject of ongoing concern and has been well developed. There were 100 papers published in 2018 and 172 papers (articles and reviews) published in 2021.

North America, Asia, and Europe were the central regions of research production. The United States undoubtedly dominated the research field of stem cell precision medicine, contributing approximately 30% of the existing publications. The leading academics with advanced research conditions and technologies might attribute such a apparent superiority. Seven of the top 10 contributing institutions were all from the United States, including Stanford University, Harvard Medical School, Icahn School of Medicine at Mount Sinai, University of Pennsylvania, University of Illinois Chicago, University of Pittsburgh, and University of Washington. Notably, the United States had active intercountry collaborations but a relatively lower MCP ratio, suggesting that research on stem cell precision medicine in America was more independent and advanced than in other countries.

Moreover, the United States retained enormous influence and had comprehensive and active scientific research cooperation with other countries, including China, Italy, Germany, England, and Japan. Remarkably, China surpassed European countries and ranked in the top 3 in terms of publications on stem cell precision medicine. Most of the high-yielding institutions were from the US, and there were only three institutions from Australia, Canada, and Italy. This information indicated that China was an Asian country with high research yields but relatively decentralized research units. These differences might be attributed to the importance the governments attached to scientific research in recent years, the availability of sufficient funding, and the rapid development of research capacity.

Journal of associated publications can reflect the overall quality of the research. The top 3 productive journals with publications relating to stem cell precision medicine were CANCERS, CELLS, and INTERNATIONAL JOURNAL OF MOLECULAR SCIENCES. The top 3 co-cited journals in this field were NATURE, CELL, and Cell Stem Cell. The top 3 journals all ranked in Q1 in Journal Citation Reports for co-citing journals, while the highest impact factor of the top 10 reached 91.253 (2020). Scientific Reports both Ranked in the top 10 as for production and co-citation. In the past five years, papers on stem cell precision medicine had not been published in top journals, but the overall journals were still distributed in the Q1 area. Generally, the quality of studies on stem cell precision medicine was considerable.

#### Background Knowledge

Highly cited papers can reflect the influential background knowledge of a field. There was no overlap between highly cited articles and highly co-cited references recently. Highly co-cited references are often the classic theories in the research field after time test. Most of the highly co-cited references in our research came from NATURE, CELL, and SCIENCE, suggesting that crucial research background knowledge in the future was likely to be found in these journals. The most cited document was “Induced Pluripotent Stem Cells for Cardiovascular Disease Modeling and Precision Medicine: A Scientific Statement From the American Heart Association” by Musunuru K in 2018. In this study, the authors stated that there were currently two ways to model cardiovascular disease using induced pluripotent stem cells: iPSC from patient-specific cells (such as skin fibroblasts) or gene editing. Cardiovascular disease models based on iPSC had covered cardiomyopathy, rhythm abnormalities, valvular and vascular diseases, ischemic heart disease, familial pulmonary hypertension, etc. However, there were still relatively intuitive differences between cardiomyocytes differentiated from iPSC and adult cardiomyocytes, reflected in cell shape, electrophysiology, calcium handling, mitochondrial physiology, and response to epinephrine signals. In terms of precision medicine, cardiovascular organoids constructed from induced stem cells played a role in screening DNA mutations of patient-specific diseases, disease development mechanisms, and treatment methods. In the future, we look forward to more large-scale production of induced pluripotent stem cells and the construction of stem cell banks for drug toxicity testing and other important directions (30).

The second document, “Disease Modeling Using 3D Organoids Derived from Human Induced Pluripotent Stem Cells” by Ho BX published in 2018 emphasized that 3D organoids from induced pluripotent stem cells could highly simulate the natural physiological environment compared with 2D models. It had advantages in constructing vascularization models and studying complex inflammation models. The author summarized the organoid disease models built by human-induced pluripotent stem cells, including the brain, liver, pancreas, small intestine, stomach, kidney, bladder, lung, retina, etc. However, there were still shortcomings in 3D organoids. The modeling technology of induced pluripotent stem cell 3D organoids was complex and required high technical requirements. Further, cells (endothelial cells, monocytes, macrophages, etc.) and cell physiology changes (leukocyte migration, monocyte differentiation, etc.) were constructed based on vascularization. The vascularization in the model was not yet complete, and wide vascular networks could not be built at this stage. Despite these, human-induced pluripotent stem cells still played an essential role in building disease models and drug screening (31).

The third one, “Organs-on-a-Chip: A Fast Track for Engineered Human Tissues in Drug Development” by Ronaldson-Bouchard K described the critical role of organ-on-a-chip in drug development. Human-induced pluripotent stem cells were involved in developing part of the organ-on-a-chip construction. The construction method of the single organoid chip included primary cells, cell lines, iPSCs, MSCs, and other cells. Multiple organ chips could be connected by static, singe-loop perfusion, recirculation, and recirculation-tissue-specific media (32). The fourth highly cited document was “Human iPSC banking: barriers and opportunities” by Huang CY in 2019, which focused on summarizing the current development of iPSC banks and introduced the cell sources, reprogramming methods, characterization methods, and operational situation of worldwide iPSC banks (33). Paik DT emphasized the vital role of patient-specific induced pluripotent stem cells in screening personalized cardiovascular drugs in “Patient and Disease-Specific Induced Pluripotent Stem Cells for Discovery of Personalized Cardiovascular Drugs and Therapeutics” in 2020 (34). This paper supplemented and enriched the one by Musunuru K in 2018. The top 5 highly cited documents highlight two key points. The construction of disease models by iPSC is essential for achieving precise treatment. Moreover, the research on cardiovascular disease models has recently received sufficient attention and development.

Co-cited references can reflect the general and influential views of background knowledge. Highly-cited references concentrated on the early stages of induced pluripotent stem cells. Studies include:

•  The original investigator Takahashi K’s research on the iPSC construction of mice and huma;
•  Yu J's improvements to the induction protocol of human iPSC;
•  The construction of brain-like and intestinal-like models;

In 2006, Takahashi K successfully reprogrammed mouse embryonic and adult fibroblasts into pluripotent stem cells for the first time through Oct3/4, Sox2, c-Myc, and Klf4 (5). The above four factors were used to successfully reprogram human skin fibroblasts into pluripotent stem cells in the following year (6). At the same time, Yu J also successfully reprogrammed human mature somatic cells into pluripotent stem cells using different combinations of factors (OCT4, SOX2, NANOG, and LIN28) (7). In 2009, Sato T constructed intestinal crypt-villus units using Lgr5 stem cells in crypts of the small intestine, which served as an important basis for subsequent organoid model construction (35). Lancaster MA constructed 3D brain organoids from human induced pluripotent stem cells in 2013, which contained developmental characteristics of the human cerebral cortex. Lancaster MA used RNA interference technology in the model for studying microcephaly (36). We believe that this information from the above studies is vital for further studies.

#### Hotspots

The information imparted by keywords is worth further exploration. In addition to search keywords, the other top 10 keywords included “cancer stem cells,” “organoids,” “regenerative medicine,” “tissue engineering,” and “gene therapy.” The number of occurrences of keywords reflects the hotspots of the research. As shown in the highly-cited documents and references, the current research hotspots focused on establishing individualized disease models, including tumor and non-tumor diseases. Gene therapy was part of the mechanistic studies in organoid models. The research on the regenerative potential of stem cells corresponded to the current stem cell replacement therapy and future tissue engineering. The construction of patient-specific complete organs based on induced pluripotent stem cells was the ultimate goal of regenerative medicine and needed more research in the future.

#### Citation Burst

The references with intense citation burst reflect the surge of citations at a specific time. We summarized the strong citation burst references to analyze the dynamics of attention on stem cell precision medicine. The document with the strongest citation burst was titled “Organogenesis in a dish: Modeling development and disease using organoid technologies” by Lancaster MA on SCIENCE, which summarized the current uses and prospects of induced pluripotent stem cells. Human-derived cells were transformed into pluripotent stem cells, and pluripotent stem cells of a specific genotype could be constructed with gene-editing tools or the patient’s genes. Furthermore, organoids were built and used for disease simulation, drug testing, and even organ replacement (37). The second strongest paper was a perspective titled “Microfluidic organs-on-chips” by Bhatia SN on Nature Biotechnology. The author conducted a comprehensive review on organs-on-chips and illuminates the basic definition (Microfluidic culture devices and Control of system parameters). Compared with the 3D static modeling method, the microfluidic organ-on-a-chip model consisted of independent but closely related subsystems. Each system could achieve fine adjustment (like cell type and location, molecular and oxygen gradients, flow levels and pattern, mechanical forcing regimens, etc.) and affect other systems through microfluidic media. Organ-on-a-Chip could control fluid flow and enhance many cells’ differentiation, function, and long-term survival. Besides, the fluid environment allowed the organ to interact with circulating cells, like blood cells and tumor cells. Cells in the model could be precisely located and integrated with fluorescence confocal microscopy, microfluorescence, TEER measurement, multi-electrode arrays for organ physiology, disease research or drug screening, and even the adsorption, distribution, metabolism, elimination, and toxicity of drugs (38).

The third was “Organoid Cultures Derived from Patients with Advanced Prostate Cancer” by Gao D on CELL. Dong Gao et al. constructed a 3D organoid model system from patient prostate cancer biopsy samples and circulating tumor cells. They used this model to simulate numerous prostate cancer molecular changes, including but not limited to TMPRSS2-ERG fusion, SPOP mutation, SPINK1 overexpression, and CHD1 loss. The author was optimistic that the model would help further prostate cancer pathogenesis research and drug screening (39). In the same year, McCracken KW et al. reported in NATURE that they de novo generated a 3D model of gastric tissue by controlling the activation of FGF, WNT, BMP, retinoic acid, and EGF signaling pathways in human pluripotent stem cells. The author discovered that H. pylori infection resulted in the virulence factor CagA action with the c-Met receptor, thus leading to epithelial proliferation (40). Burridge PW published his research in Nature Methods to optimize the cardiac differentiation culture conditions of human-induced pluripotent stem cells, using a medium consisting of the basal medium RPMI 1,640, L-ascorbic acid 2-phosphate, and rice-derived recombinant human albumin. This culture condition yielded 95% tnnt2 + cardiomyocytes in the small molecule-based differentiation induction and achieved a 1:100 productivity in 11 human induced pluripotent stem cell lines (41).

From the citation burst references, we can also see that stem cell precision medicine has focused on creating and applying 3D, patient-specific disease models involving prostate cancer, stomach, and heart in the last five years. In the future, the establishment of multi-organ and multi-disease 3D organoid models will remain an important development direction for the application of induced pluripotent stem cells.

## Limitations

Our study was based on the tools of bibliometrics, in which the error of document statistics was hard to avoid. Our literature search was limited to WoSCC, and the analysis based on one data source might lead to biased results. The bibliometric method is closely related to time. The results of the analysis vary significantly with the choice of time span. Our study was limited to the last five years, which meant that our results were time-bound, and we could not fully understand the disease. Meanwhile, the analysis results are not suitable for continued use in the future. Especially, the burst analysis is a relatively niche approach to literature analysis in CiteSpace that calculates the surge of citations in a period. This analysis results reflect the “speed” of citations rather than the “number” of citations. It represents those papers which have attracted attention at some point, and the relevant papers may be previously published studies which have recently attracted attention. Furthermore, the bibliometric analysis is based on a certain form of publication. The result is that other forms of study or unpublished studies are not considered in our research, which may influence the authority of our conclusions. In addition, our study can also be affected by changes in journal titles. Nevertheless, our study was built on the most extensive database and had well-developed and optimized tools, which to a large extent, could be a future reference for clinicians and researchers studying stem cell precision medicine.

## Conclusion

In the present study, we conducted a comprehensive analysis of the existing research in stem cell precision medicine from 2018 to 2022, applying bibliometrics tools. Globally the study of stem cell precision medicine was undergoing a stable development over the last five years, and the year 2021 peaked its literature quantity. The United States was the most influential country regarding the number of research production and had active academic cooperation with countries, including Italy, China, Germany, and other countries. Notably, Asian countries led by China had recently taken a significant role in this field. CANCERS (IF 6.639) was the journal with the highest production of publications in stem cell precision medicine. BIOMATERIALS had the highest IF (12.479) among the top 10 productive journals. The most cited document on stem cell precision medicine was “Induced Pluripotent Stem Cells for Cardiovascular Disease Modeling and Precision Medicine: A Scientific Statement From the American Heart Association,” published by Musunuru K in 2018 with a local citation of 9 times. Takahashi K et al. published the study “Induction of pluripotent stem cells from adult human fibroblasts by defined factors,” in CELL, which had been co-cited with other references for 91 times recently. “precision medicine” (*n* = 89, 12.64%), “personalized medicine” (*n* = 72, 10.23%), “stem cells” (*n* = 43, 4.40%), “induced pluripotent stem cells” (*n* = 41, 5.82%), “cancer stem cells” (*n* = 31, 4%) and “organoids” (*n* = 26, 3.69%) co-occurred closely and were high-frequency keywords. They represented the disciplinary basis of stem cell precision medicine. The content of the top three citation burst documents focused on the current and future uses of induced pluripotent stem cells, giving optimism for their use in disease modeling, drug trials, and even organ replacement. These areas were relatively popular directions for stem cell precision medicine and worth further research in the future.

## Data Availability

The datasets used and analysed during the current study are available from the corresponding author on reasonable request.
